# The Z-nucleic acid sensor ZBP1 in health and disease

**DOI:** 10.1084/jem.20221156

**Published:** 2023-07-14

**Authors:** Jonathan Maelfait, Jan Rehwinkel

**Affiliations:** 1 VIB-UGent Center for Inflammation Research, Ghent, Belgium; 2 Department of Biomedical Molecular Biology, Ghent University, Ghent, Belgium; 3 Medical Research Council Human Immunology Unit, Medical Research Council Weatherall Institute of Molecular Medicine, Radcliffe Department of Medicine, University of Oxford, Oxford, UK

## Abstract

Nucleic acid sensing is a central process in the immune system, with far-reaching roles in antiviral defense, autoinflammation, and cancer. Z-DNA binding protein 1 (ZBP1) is a sensor for double-stranded DNA and RNA helices in the unusual left-handed Z conformation termed Z-DNA and Z-RNA. Recent research established ZBP1 as a key upstream regulator of cell death and proinflammatory signaling. Recognition of Z-DNA/RNA by ZBP1 promotes host resistance to viral infection but can also drive detrimental autoinflammation. Additionally, ZBP1 has interesting roles in cancer and other disease settings and is emerging as an attractive target for therapy.

## Teaser

ZBP1 is an innate immune sensor for double-stranded nucleic acids adopting the Z conformation. Z-RNA/DNA is generated during viral infections and in autoinflammation and cancer. By inducing regulated cell death and proinflammatory signaling, ZBP1 plays multifaceted roles in disease pathology.

## Introduction

Nucleic acids not only store genetic information and mediate gene expression but are also signals that trigger activation of the immune system. This process—known as “nucleic acid sensing” or “nucleic acid immunity”—involves numerous germline-encoded innate immune receptors for DNA and RNA (Bartok and Hartmann, 2020). One such receptor is Z-DNA binding protein 1 (ZBP1, also known as DLM-1 or DAI). ZBP1 senses double-stranded (ds) DNA and RNA that adopt, or are prone to adopt, a left-handed, double-helical structure known as “Z.” Much work over the last few years has led to the concept whereby atypical Z-DNA/RNA is perceived by ZBP1 as a molecular signature of infection and in autoinflammation. Once activated by Z-DNA/RNA, ZBP1 induces apoptosis, necroptosis, and pyroptosis, three forms of regulated cell death, and activates inflammatory signaling via NF-κB. ZBP1 thereby plays important roles in many disease settings, ranging from viral infection and inflammation to cancer, and efforts to target ZBP1 are underway. Here, we summarize the evidence that has led to these conclusions, discuss unexpected recent observations, and highlight open questions that require attention to fully harness ZBP1’s therapeutic potential.

## ZBP1, a Z-DNA/RNA binding protein

ZBP1 was first cloned from mouse macrophages as a protein called DLM-1 that is induced by IFN-γ and may have an antitumor function (Fu et al., 1999). It is now well established that not only IFN-γ but also type I IFNs (including IFN-α and IFN-β) strongly induce the expression of ZBP1. Shortly after its initial description, Rich and colleagues revealed that ZBP1 contains two Zα domains (Schwartz et al., 2001). This small domain belongs to the family of winged helix-turn-helix motifs and is found in only a few proteins including mammalian ADAR1, fish PKZ, and viral proteins such as vaccinia virus E3 (Athanasiadis, 2012). Zα domains bind specifically to dsDNA and dsRNA in the unusual Z conformation. This left-handed double helix was first discovered in 1979 (Wang et al., 1979) but biological roles have remained somewhat enigmatic until recently. Typically, dsDNA and dsRNA adopt the B and A conformations, respectively, right-handed double helices. These conventional and well-known conformations are energetically favored under physiological conditions. However, high salt concentration or binding to proteins containing Zα domains can stabilize dsDNA/RNA in the Z conformation in regions containing repeating purine–pyrimidine units. In contrast to A and B, the Z conformation is a left-handed double helix with a zig-zag-shaped phosphodiester backbone, hence the designation Z. Additional distinguishing features include alternating syn*-* and anti-conformations of the nucleobases in Z-DNA/RNA. We refer the reader to excellent recent reviews on Z nucleic acids for further information on their structure and properties (Herbert, 2019; Krall et al., 2023; Nichols et al., 2023).

Following the description of a dsDNA sensing pathway surveying the cytosol of mammalian cells (Ishii et al., 2006; Stetson and Medzhitov, 2006), ZBP1 was proposed as an apical sensor for B-dsDNA and to trigger type I IFN production; DNA-dependent activator of IFN-regulatory factors (DAI) was coined as a new name for the protein (Takaoka et al., 2007). However, subsequent knockout studies (Ishii et al., 2008) and the discovery of cGAS (Gao et al., 2013) led to the now widely accepted notion that the cGAS-STING pathway is the main driver of cytosolic B-dsDNA–induced type I IFN (Ablasser and Chen, 2019). Accordingly, ZBP1 is now the standard nomenclature and most widely used name, although it is noteworthy that another, unrelated protein, zipcode binding protein 1, shares the same abbreviation.

**Figure 1.**
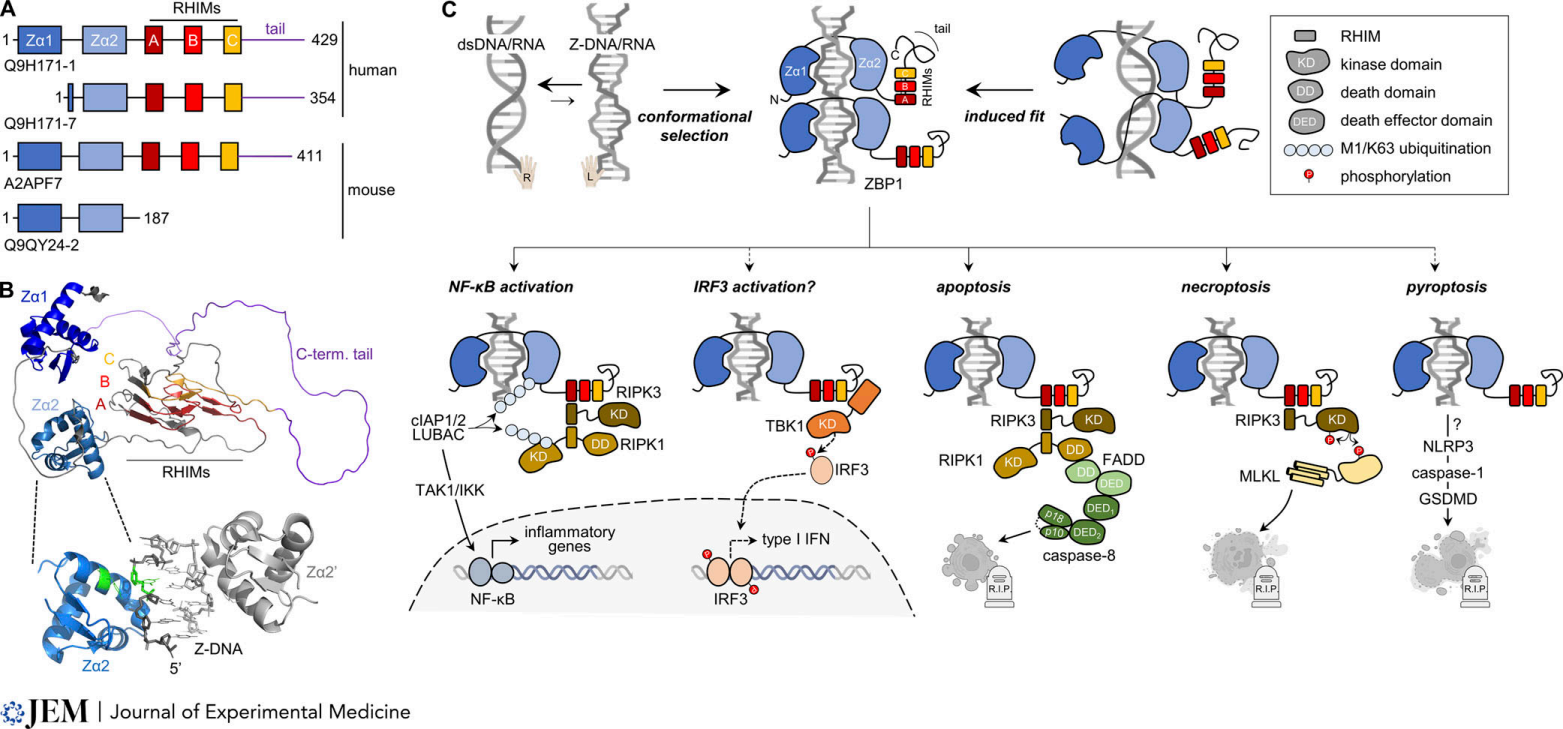
**ZBP1 domain structure and signaling. (A)** Schematic overview of the domain architectures of the two major human and mouse isoforms of ZBP1 detectable by Western blot. UniProt identifiers are shown below each structure. Humans express a second isoform termed ZBP1-S, which lacks exon 2 encoding the first Zα domain, while mice express a second isoform only encompassing the two Zα domains. The role of these isoforms remains mostly unknown; human ZBP1-S is thought to induce a MAVS-dependent type I IFN response following recognition of TERRA transcripts (see “The role of ZBP1 in cancer”). **(B)** Top: AlphaFold prediction of the structure of human ZBP1. Both Zα domains form a winged helix-turn-helix structure that enables them to specifically bind to Z-RNA/DNA. The β-sheets of the three consecutive RHIMs stack in an amyloidal structure on top of each other. Bottom: Z-DNA/Zα2 crystal structure (PDB 3EYI). Two Zα2 domains bind in an antiparallel fashion to Z-DNA with a 5 bp footprint. Tyr^145^ located in the third α-helix of the Zα domain forms the only base contact with syn-dG (both shown in green) through a CH-π interaction. **(C)** Schematic of the different signaling outcomes of ZBP1 activation. ZBP1 may interact with Z-DNA/RNA in two manners. Due to the chemical equilibrium, a fraction of right-handed dsDNA/RNA adopts the Z-conformation. These molecules are then “trapped” in the left-handed Z conformation through conformational selection by the Zα domains of ZBP1. Alternatively, Z-prone nucleic acids may be actively “pushed” into the Z conformation by ZBP1’s Zα domains (induced fit). Activated ZBP1 then induces downstream signaling via its RHIMs. During NF-κB activation, the first RHIM of ZBP1 (RHIM-A) mediates binding to RIPK1 and RIPK3 through homotypic RHIM interactions. Both K63- and M1-linked ubiquitin chains are then attached to ZBP1 and RIPK1 by the K63-specific ubiquitin E3 ligases cIAP1 and cIAP2 and the linear ubiquitin chain assembly complex (LUBAC), which mediates M1 ubiquitination. These ubiquitin chains then recruit the TAK1 and IKK kinase complexes, which activate the NF-κB transcription factor resulting in inflammatory gene expression. ZBP1 has also been reported to activate the IRF3 transcription factor via TBK1 to induce type I IFN expression. The detailed mechanisms that are involved in this process remain to be described. The ZBP1/RIPK3/RIPK1 complex can also induce apoptosis after recruitment and activation of caspase-8 via FADD. Like TNF signaling, this may occur when ubiquitination of the ZBP1/RIPK3/RIPK1 complex is perturbed or when the expression of anti-apoptotic proteins such as cFLIP is downregulated. When caspase-8 activation is inhibited, ZBP1 activation results in the formation of a necrosome whereby RIPK3 phosphorylates and activates the pore-forming protein MLKL, resulting in necroptosis. At least in mouse cells, this occurs independently of RIPK1. In macrophages, ZBP1 stimulates activation of the NLRP3 inflammasome through a mechanism that has not yet been clearly defined, resulting in activation of caspase-1, which cleaves and activates the pore-forming protein GSDMD, resulting in pyroptosis. Figures of DNA, RNA, and dying cells were created with BioRender.com.

In addition to the two Zα domains (Zα1 and Zα2) in its N-terminal portion, ZBP1 has three centrally located RIP-homotypic interaction motifs (RHIMs; Fig. 1, A and B). These motifs mediate interaction with other RHIM-containing proteins and regulate downstream signaling (Sun et al., 2002; Fig. 1 C). Apart from ZBP1, RHIMs are found in TRIF, an adaptor protein for Toll-like receptor 3 (TLR3) and TLR4, and the receptor-interacting protein kinases (RIPK)1 and RIPK3. These kinases mediate signaling downstream of ZBP1 and instigate distinct cellular responses. This includes activation of NF-κB that then induces the expression of proinflammatory cytokines and chemokines. Mechanistically, this involves RIPK1 and RIPK3 recruitment to active ZBP1 and their ubiquitination (Hayashi et al., 2010; Kaiser et al., 2008; Peng et al., 2022; Rebsamen et al., 2009). RIPK1 and RIPK3 further cooperate to induce caspase-8–mediated apoptosis downstream of ZBP1 (Kuriakose et al., 2016; Thapa et al., 2016). ZBP1-activated RIPK3 also phosphorylates the pseudokinase MLKL, which in turn attacks the plasma membrane by forming pores, ultimately killing the cell (Upton et al., 2012). This form of regulated cell death is known as necroptosis and releases cellular contents, rendering it immunogenic (Vandenabeele et al., 2023). ZBP1 has also been suggested to induce pyroptosis, another form of regulated inflammatory cell death, by activating the formation of inflammasomes (Kuriakose et al., 2016). Finally, as discussed below, ZBP1-dependent type I IFN induction—albeit not triggered by B-dsDNA—has recently been reported in some settings of viral infection and autoinflammation (Fig. 1 C).

## ZBP1 in antiviral defense

### ZBP1 induces antiviral cell death

In 2012, Upton and colleagues were the first to assign a clear antiviral function to ZBP1 (Upton et al., 2012). Mechanistically, ZBP1 activation restricts replication of murine cytomegalovirus (MCMV), a member of the Herpesviridae, by inducing host cell necroptosis. This occurs through the recruitment and activation of RIPK3 to active ZBP1. At least in the mouse system and in contrast to TNF-mediated necroptosis, ZBP1-induced necroptotic signaling proceeds independently of RIPK1 and its kinase activity (Upton et al., 2012). Necroptosis is normally prevented by the viral inhibitor of RIP activation (vIRA), encoded by MCMV *M45* (Upton et al., 2010). M45 is a catalytically inactive homolog of the large subunit of ribonucleotide reductase (Brune et al., 2001) and contains a RHIM at its N-terminus, which interferes with the assembly of a ZBP1–RIPK3 necroptotic signaling complex (Upton et al., 2012). Later, other large DNA viruses including herpes simplex virus-1 (HSV-1), varicella zoster virus (VZV), and the vaccinia poxvirus were found to induce ZBP1-mediated host cell death, which halts viral replication (Guo et al., 2018; Koehler et al., 2017; Steain et al., 2020). HSV-1 and possibly also HSV-2 inhibit ZBP1-mediated necroptosis through the *UL39*-encoded RHIM containing ICP6 and ICP10 proteins, homologs of M45 (Guo et al., 2015; Guo et al., 2018; Huang et al., 2015). VZV contains a RHIM within the triplex capsid subunit 1 protein encoded by *ORF20* to inhibit cell death downstream of ZBP1 (Steain et al., 2020). Vaccinia virus is currently not known to encode a RHIM protein. Instead, the viral E3 protein, encoded by the *E3L* gene, contains a Zα domain and blocks ZBP1 signaling by sequestration of Z-RNA (Koehler et al., 2021).

The antiviral effects of ZBP1 are also well-documented for influenza A virus (IAV), a negative-stranded single-stranded (ss) RNA virus. As opposed to infections with large DNA viruses, IAV-mediated ZBP1 activation induces both RIPK3-RIPK1-caspase-8–dependent apoptosis and RIPK3-MLKL–mediated necroptosis (Kuriakose et al., 2016; Thapa et al., 2016). Blockade of both cell death pathways is required to fully prevent cell death following IAV infection. On a per-cell basis, apoptosis and necroptosis induction downstream of ZBP1 have been suggested to be mutually exclusive events (Shubina et al., 2020). The decision to activate one or the other cell death modality may occur stochastically or depend on yet-to-be defined variables. At least in cultured bone marrow–derived macrophages (BMDMs), IAV-induced ZBP1 activation supports yet another cell death pathway: pyroptosis, which is characterized by the maturation of the inflammatory cytokines IL-1β and IL-18 into their biologically active forms (Kuriakose et al., 2016). Whether this occurs downstream of ZBP1-mediated apoptosis and necroptosis through secondary activation of the NLRP3 inflammasome (Lei et al., 2023a) or in parallel to apoptosis and necroptosis (Zheng et al., 2020) remains controversial. Differences between these studies may relate to whether and how the expression of inflammasome components is primed and perhaps also to viral doses used.

It is important to note that IAV does not produce designated proteins that interfere with either ZBP1-initiated apoptosis or necroptosis. In contrast, herpesviruses and poxviruses encode antagonists of caspase-8 activation and proteins that block RIPK3-mediated necroptosis (Verdonck et al., 2022). The studies wherein MCMV, HSV-1, and vaccinia virus selectively induce necroptosis after ZBP1 activation were performed using viral strains that lacked RIPK3 antagonistic activity but retained expression of caspase-8 inhibitors. In fact, caspase-8 inhibition further sensitizes cells to RIPK3-MLKL–mediated necroptosis (Orning and Lien, 2021), explaining why ZBP1 activation results in overtly necroptotic phenotypes in these infectious settings. Indeed, infection with VZV, which does not express any known inhibitors of caspase-8, triggers apoptosis downstream of ZBP1 in human cells (Steain et al., 2020), and a wild-type HSV-1 strain induces both ZBP1-mediated apoptosis and necroptosis in mouse astrocytes (Jeffries et al., 2022). It is not clear why the HSV-1–encoded inhibitors of cell death fail to maintain astrocyte viability. The fact that in mouse cells ICP6 functions as an inducer rather than inhibitor of necroptosis (Guo et al., 2015; Huang et al., 2015; Wang et al., 2014) further complicates the interpretation of this study. Activation of ZBP1 by RNA and DNA virus infection thus activates both apoptotic and necroptotic signaling cascades to cut short viral replication. Large dsDNA viruses in particular have evolved multiple strategies to evade immune recognition by ZBP1 (Table 1).

**Table 1.** Viruses recognized by ZBP1

Virus	Family	Proposed antiviral function(s)	Role(s) in in vivo mouse models	Viral ZBP1 antagonist	ZBP1 agonist	References
MCMV	Herpesviridae (large dsDNA)	Necroptosis	Restriction of viral replication Protective	M45 (vIRA) inhibits necroptosis	RNA	Jiao et al., 2020; Maelfait et al., 2017; Sridharan et al., 2017; Upton et al., 2012
UV-inactivated human cytomegalovirus		Contribution to type I IFN response	n.a.	UL36 potentially inhibits necroptosis	n.d.	DeFilippis et al., 2010
HSV-1		Necroptosis and apoptosis (and pyroptosis in BMDMs) Contribution to type I IFN response and inflammatory cytokine production Potential inhibition of ICP0-mediated degradation of IFI16	Restriction of viral replication	ICP6 (UL39) inhibits necroptosis	n.d.	Furr et al., 2011; Guo et al., 2018; Jeffries et al., 2022; Lee et al., 2021; Pham et al., 2013; Takaoka et al., 2007
HSV-2		Necroptosis? Contribution to type I IFN response	n.d.	ICP10 (UL39) potentially inhibits necroptosis	n.d.	Triantafilou et al., 2014
VZV		Apoptosis	n.d.	Triplex capsid subunit 1 (ORF20) inhibits caspase-dependent cell death	n.d.	Steain et al., 2020
Vaccinia virus	Poxviridae (large dsDNA)	Necroptosis	Restriction of viral replication Protective	E3 (E3L) sequesters Z-RNA	Z-RNA	Koehler et al., 2017, 2021
IAV	Orthomyxoviridae (−ssRNA)	Apoptosis and necroptosis (and pyroptosis in BMDMs) Contribution to inflammatory cytokine production	Restriction of viral replication Protective or contribution to immunopathology	n.d.	Viral Z-RNA (e.g., subgenomic RNA)	Karki et al., 2022; Kuriakose et al., 2016; Momota et al., 2020; Thapa et al., 2016; Zhang et al., 2020
SARS-CoV-2	Coronaviridae (+ssRNA)	Apoptosis and necroptosis (and pyroptosis in BMDMs) Contribution to inflammatory cytokine production	No effect on viral replication Causes immunopathology	n.d.	Viral Z-RNA	Li et al., 2023; Peng et al., 2022
MHV		Apoptosis and necroptosis (and pyroptosis in BMDMs)	Causes immunopathology following IFN-β treatment	n.d.	n.d.	Karki et al., 2022
Zika virus	Flaviviridae (+ssRNA)	IRF1-dependent upregulation of IRG1 resulting in a metabolic antiviral state in neurons	Restriction of viral replication, better survival	n.d.	n.d.	Daniels et al., 2019; Rothan et al., 2019
West Nile virus	Flaviviridae (+ssRNA)	n.d.	Restriction of viral replication, better survival	n.d.	n.d.	Rothan et al., 2019

Viruses known to be detected by ZBP1 are shown, along with viral families and proposed antiviral functions of ZBP1. Where these have been determined, in vivo roles of ZBP1, viral ZBP1 antagonists, and nucleic acid agonists of ZBP1 are given. n.d., not determined; n.a., not applicable.

### Viral agonists of ZBP1

Immunostaining with antibodies raised against Z-DNA and with crossreactivity against Z-RNA shows that Z-RNA accumulates in the nucleus of IAV-infected cells (Zhang et al., 2020). This results in nuclear activation of ZBP1 and MLKL, causing breakdown of the nuclear envelope. This nuclear form of necroptosis is proposed to be particularly inflammatory by the release of immunostimulatory proteins such as IL-33 and HMGB1, thereby promoting neutrophil-driven lung pathology (Zhang et al., 2020). From this study, it is not yet clear how caspase-8–mediated apoptotic signaling disseminates from the nucleus. As opposed to IAV, infection with vaccinia virus and SARS-CoV-2 results in Z-RNA accumulation in the cytosol, coinciding with their cytosolic replication cycles (Koehler et al., 2021; Li et al., 2023). The vaccinia E3 protein has a surprising role in ZBP1 regulation: its C-terminal A-form dsRNA-binding domain promotes Z-RNA formation while its N-terminal Zα domain competes with ZBP1 for the very agonist it induces. ZBP1 activation by vaccinia, therefore, requires the presence of an intact E3 dsRNA-binding domain and a dysfunctional Zα domain (Koehler et al., 2021). Activation of ZBP1 by other DNA viruses including MCMV and HSV-1 requires newly transcribed viral RNA (Guo et al., 2018; Maelfait et al., 2017; Sridharan et al., 2017), further supporting the idea that DNA viruses activate ZBP1 through Z-RNA production.

Thus far, the precise nature of the ligands that activate ZBP1 remains poorly defined. In the case of IAV, genomic RNA and in particular defective viral genomes immunoprecipitate with ZBP1 (Thapa et al., 2016; Zhang et al., 2020), and ZBP1 concentrates around viral ribonucleoprotein particles (Kesavardhana et al., 2017). The dsRNA panhandle region formed by pairing of the 5′ and 3′ ends of viral (sub)genomic RNA may constitute the IAV-induced ZBP1 agonist. In the case of DNA viruses, dsRNA molecules formed by pairing of overlapping transcripts derived from opposing genomic DNA strands may adopt Z-conformations. Why these virus-derived RNA molecules are stabilized in the Z-form is not known and at least two non-mutually exclusive scenarios are possible. First, RNA and DNA virus infection greatly increase intracellular dsRNA concentrations (Son et al., 2015; Weber et al., 2006). Given the fact that a small fraction of these molecules will adopt the Z conformation due to the chemical equilibrium between both the A and Z conformers (Krall et al., 2023), more Z-RNA will be present as well. The second possibility is a shift in the chemical equilibrium between A- and Z-RNA. The Z-transition process may be facilitated by nucleotide modifications, changes in relative sequence abundancies, mechanical strain (Krall et al., 2023), and active participation of ZBP1 in the Z-transition process (Ha et al., 2008; Kim et al., 2011a; Kim et al., 2011b).

### Cell death–independent activities of ZBP1 during virus infection

Apart from inducing cell death, ZBP1 also contributes to the induction of inflammatory genes and the type I IFN response. This has been documented for a number of DNA and RNA viruses including human cytomegalovirus (DeFilippis et al., 2010), HSV-1 (Furr et al., 2011; Pham et al., 2013; Takaoka et al., 2007), HSV-2 (Triantafilou et al., 2014), IAV (Kuriakose et al., 2016), SARS-CoV-2 (Li et al., 2023; Peng et al., 2022), and Zika virus (Daniels et al., 2019; Table 1). Ectopic expression of ZBP1 spontaneously triggers NF-κB activation resulting in inflammatory gene expression in the absence of cell death induction, showing that induction of transcription can be functionally separated from cell death signaling (Peng et al., 2022). The function of ZBP1 in transcriptional responses in more complex scenarios including natural infections will likely be more difficult to dissect given the redundancy with other ubiquitously expressed nucleic acid sensors that specialize in the induction of transcription of antiviral genes.

### In vivo functions of ZBP1

Studies in *Zbp1* knockout mice or knock-ins expressing a ZBP1 protein that is unable to interact with Z-RNA/DNA clearly demonstrate the physiological role of ZBP1 in suppressing replication of MCMV (Jiao et al., 2020; Maelfait et al., 2017; Upton et al., 2012), vaccinia virus (Koehler et al., 2017), HSV-1 (Guo et al., 2018), IAV (Kuriakose et al., 2016; Thapa et al., 2016), and the neurotropic West Nile and Zika flaviviruses (Daniels et al., 2019; Rothan et al., 2019; Table 1). Zika virus engages an atypical cell death–independent ZBP1 signaling pathway involving IRF1-dependent transcriptional upregulation of ACOD1 (also known as IRG1), a mitochondrial metabolic enzyme that produces itaconate from the Krebs cycle intermediate cis-aconitate (Daniels et al., 2019). Itaconate inhibits viral replication through inhibition of succinate dehydrogenase, an enzyme complex of the electron transport chain and Krebs cycle. This pathway depends on both the kinase activity of RIPK1 and RIPK3 and operates uniquely in neurons. How exactly inhibition of succinate dehydrogenase restricts Zika virus infection in this cell type is not known.

Better viral clearance does not, however, always correlate with better disease outcome. For example, the in vivo consequence of ZBP1 activation following IAV is variable with some studies reporting worse (Karki et al., 2022; Thapa et al., 2016) and others documenting better survival (Kuriakose et al., 2016) of *Zbp1* knockout mice. These discrepancies may be caused by experimental parameters that determine the severity of disease such as the viral strain, infectious dose, genetic background of *Zbp1* knockout mice (Koehler et al., 2020), and the route of infection. Indeed, *Zbp1* knockouts are less resistant to IAV infection following intranasal delivery, which causes slower and milder disease progression. Conversely, ZBP1-deficient mice infected via the intratracheal route, which causes more severe and acute disease, tolerate infection better and develop less immunopathology (Momota et al., 2020). This study also demonstrated a critical role for ZBP1 in the release of the alarmin IL-1α attracting neutrophils to the lung, which may aid both viral clearance and drive tissue damage. A contribution of ZBP1 to immunopathology is also seen during infection with SARS-CoV-2 or mouse hepatitis virus (MHV), two members of the positive-stranded ssRNA Coronaviridae. In the case of SARS-CoV-2, ZBP1 promotes proinflammatory cytokine production, recruitment of monocytes and neutrophils, and lung damage without affecting viral loads (Li et al., 2023). In the case of MHV, upregulation of ZBP1 expression by therapeutic administration IFN-β decreased survival of MHV-infected mice, probably by inducing cell death–mediated immunopathology (Karki et al., 2022).

In sum, ZBP1 activation by viral Z-RNA suppresses DNA and RNA virus infection through cell death–dependent and –independent mechanisms. These antiviral functions of ZBP1 and viral evasion strategies are summarized in Table 1.

## ZBP1 and autoinflammation

**Figure 2.**
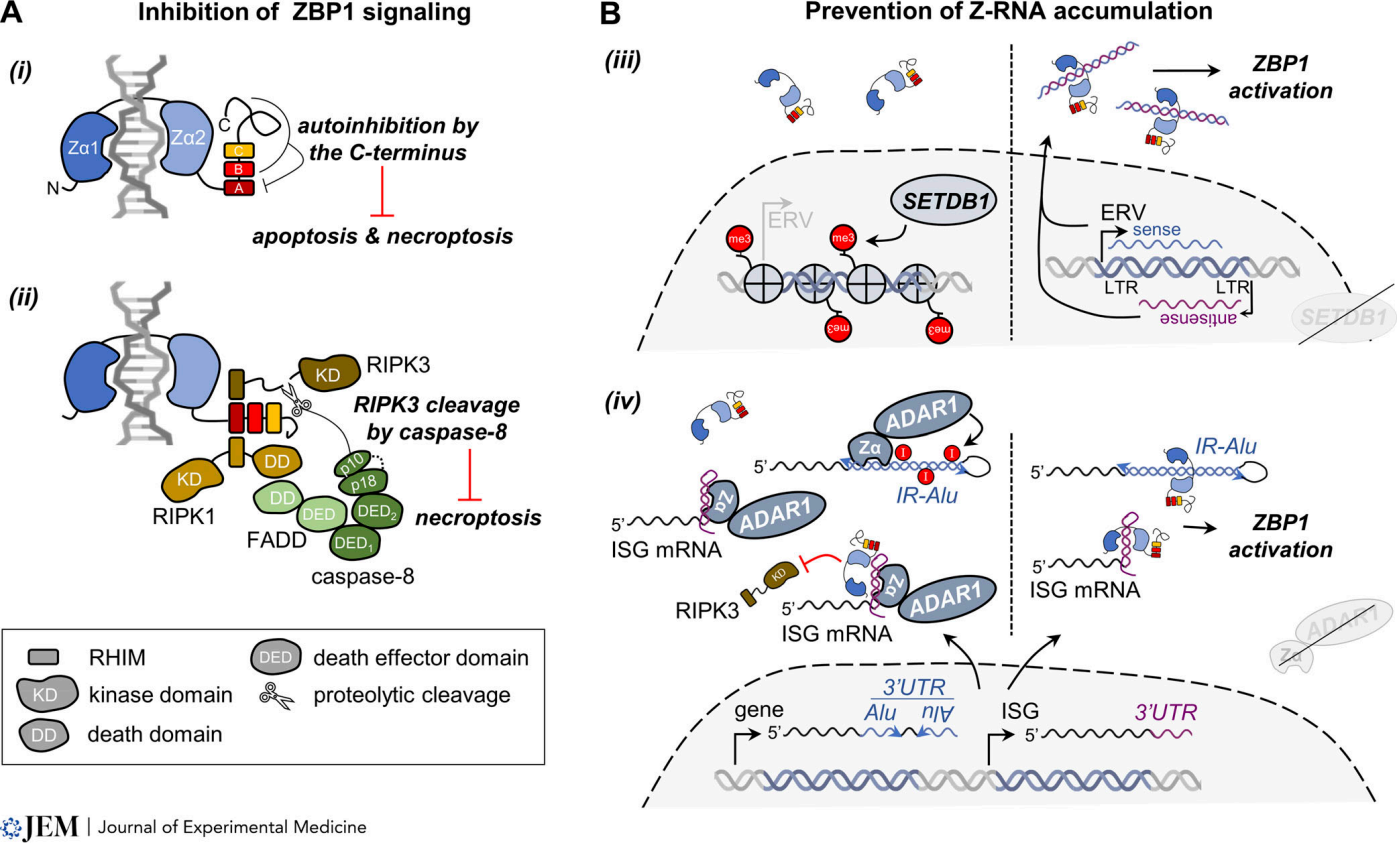
**Negative regulation of ZBP1 activation. (A)** (i) Apoptosis and necroptosis induction downstream of mouse ZBP1 is inhibited by the RHIM-B/C containing C-terminus. The mechanism by which this occurs is not yet clear. (ii) Recruitment and activation of caspase-8 by RIPK1 and FADD to active ZBP1 prevents necroptosis. Caspase-8 proteolytically cleaves mouse RIPK3 after Asp^333^ thereby releasing its kinase domain from the signaling complex. A similar mechanism may be involved in restraining ZBP1-mediated necroptosis in human cells. **(B)** (iii, left) Trimethylation (me3) of histone H3 Lys9 by SETDB1 at loci coding for endogenous retroviruses (ERVs) suppresses ERV transcription. (iii, right) In the absence of SETDB1, overlapping sense and antisense transcripts are transcribed from the bidirectional long-terminal repeat (LTR) promotors of ERVs. These transcripts form dsRNA, which may adopt the Z-conformation and activate ZBP1 in the cytosol. (iv, left) Sequestration of Z-RNA by the Zα domain of ADAR1 prevents ZBP1 activation. Alternatively, mutual binding of ADAR1 and ZBP1 to Z-RNA prevents RIPK3 recruitment to ZBP1. ADAR1 binds Z-RNA formed by foldback of IR-*Alu*s found in the 3′ UTRs of many genes or short complementary sequences within the 3′ UTRs of IFN-stimulated genes (ISGs). The Zα domain of ADAR1 further enhances adenosine-to-inosine (A-to-I) editing of IR-*Alu*s, which prevents recognition of these structures by ZBP1. (iv, right) Loss of ADAR1 function results in the accumulation of unedited (Z-form) dsRNA inside the cytosol and the recognition of Z-RNAs by ZBP1, resulting in its activation. Figures of DNA and RNA were created with BioRender.com.

Multiple studies show that activation of ZBP1—in addition to antiviral immunity—promotes the development of sterile inflammation. Evidence supporting a role for ZBP1 in autoinflammation initially came from genetic mouse studies aimed at determining the physiological function of the RHIM of RIPK1 (Lin et al., 2016; Newton et al., 2016). Mice that express a RHIM-mutant RIPK1 protein die perinatally due to excessive necroptosis induction caused by spontaneous ZBP1 activation. It is noteworthy that the dominant role of ZBP1 in this model differs from full *Ripk1* knockouts, wherein both TNF-induced apoptosis and ZBP1-mediated necroptosis contribute to postnatal lethality (Ingram et al., 2019; Newton et al., 2016). In addition to ZBP1, TRIF-mediated necroptosis, which in some cell types also proceeds independently from RIPK1 (Kaiser et al., 2013; Kim and Li, 2013), underlies some of the inflammatory phenotypes of RHIM-mutant *Ripk1* mice (Jiao et al., 2020; Newton et al., 2016). Activation of ZBP1 in RIPK1-deficient cells depends on its type I/II IFN–induced transcription (Ingram et al., 2019; Yang et al., 2020) and binding of ZBP1 to endogenous Z-nucleic acids, the precise identity of which remains unknown (Devos et al., 2020; Jiao et al., 2020; Kesavardhana et al., 2020). How the RHIM of RIPK1 inhibits ZBP1 necroptotic signaling is not entirely understood. This may involve recruitment of caspase-8 to the active ZBP1 complex via FADD, enabling caspase-8–mediated cleavage of mouse RIPK3 after D333 (D228 in human RIPK3) between the kinase domain and the RHIM resulting in its inactivation (Fig. 2 A; Yang et al., 2020). The physiological importance of the RIPK1/FADD/caspase-8 brake on ZBP1 is evident from intestinal epithelium-specific *Fadd* or *caspase-8* deficient mice, which develop ZBP1-induced, necroptosis-driven colon inflammation (Jiao et al., 2020; Schwarzer et al., 2020). A recent preprint describes that C-terminal truncation of ZBP1 preserving only the two Zα domains and the first RHIM renders ZBP1 constitutively active (Körner et al., 2023 *Preprint*). Overexpression of this ZBP1 molecule in the epidermis of mice causes autoinflammation by engaging both RIPK1-FADD-caspase-8–mediated apoptosis and RIPK3-MLKL–dependent necroptosis. Apart from demonstrating that forced ZBP1 activation in vivo causes both apoptosis and necroptosis, this study also reveals the presence of autoinhibitory functions of the second and third RHIM and C-terminal portion of ZBP1 (Fig. 2 A).

Thus far, two enzymes, SETDB1 and ADAR1, have been shown to control the accumulation of endogenous ZBP1 agonists. SETDB1 is a histone H3 methyltransferase involved in silencing the expression of endogenous retroviruses (Matsui et al., 2010). Removal of SETDB1 in intestinal epithelium causes ZBP1-mediated intestinal inflammation (Wang et al., 2020). DsRNA accumulation in SETDB1 deficient cells and copurification of endogenous retroviral sequences with ZBP1 strongly indicate that Z-RNA formation of reactivated endogenous retroviral RNA triggers ZBP1 in this context (Fig. 2 B). Apart from ZBP1, ADAR1 is the only mammalian protein containing a Zα domain. ADAR1 catalyzes the conversion of adenosines into inosines specifically within dsRNA. This process, called A-to-I editing, suppresses accumulation of endogenous dsRNA and spontaneous activation of dsRNA sensors including MDA5 (Ahmad et al., 2018; Mannion et al., 2014; Pestal et al., 2015). Loss-of-function of ADAR1 causes the type I IFN–mediated inflammatory disease Aicardi-Goutières syndrome (Rice et al., 2012). Mice that recapitulate the compound heterozygous state of patients (i.e., one Zα domain mutant *ADAR1* allele combined with a second *ADAR1* null allele) develop lethal autoinflammation, albeit with varying penetrance depending on the type of Zα domain mutation (de Reuver et al., 2021; Jiao et al., 2022; Liang et al., 2023; Maurano et al., 2021). ZBP1 drives fatal pathology in these animals showing that ADAR1 is a negative regulator of ZBP1 (de Reuver et al., 2022; Hubbard et al., 2022; Jiao et al., 2022). Although ZBP1 activation in ADAR1-deficient cells causes both caspase-8–mediated apoptosis and MLKL-dependent necroptosis, the genetic deletion of both pathways in Zα domain mutant mice does not rescue lethal inflammation or even worsens the phenotype. Removal of caspase-8 and/or MLKL in these mice possibly unleashes ZBP1-driven inflammatory gene expression (Hubbard et al., 2022) or other yet-to-be-defined innate immune pathways in the highly type I IFN–mediated inflammatory context of ADAR1 deficiency, resulting in failure to rescue the phenotype. Interestingly, ZBP1 also contributes to the type I IFN–dependent gene expression in ADAR1 Zα domain mutant mice (de Reuver et al., 2022; Hubbard et al., 2022; Jiao et al., 2022); however, it is not clear if this occurs directly downstream of ZBP1 or is an indirect consequence of ZBP1-mediated cell death.

Hemizygous expression of Zα domain mutant ADAR1 results in impaired A-to-I editing of short interspersed nuclear elements including *Alu* elements found in the 3′ UTR of many messenger RNAs (de Reuver et al., 2022; Jiao et al., 2022). Base pairing of two inversely oriented *Alu*s (IR-*Alu*s) located within the same transcript form a potential source of endogenous ZBP1 agonists. Indeed, transfection of in vitro transcribed IR-*Alu*s into cells activated ZBP1 (de Reuver et al., 2022). The Zα domain of ADAR1 thus stimulates A-to-I editing of IR-*Alu*s, resulting in the destabilization of these structures and preventing recognition by ZBP1, similar to the inhibitory mechanism of ADAR1 toward MDA5 (Ahmad et al., 2018). Additionally, sequestration of short Z-RNA prone sequences and inverted short interspersed nuclear elements within the 3′ UTR of IFN-stimulated genes by the Zα domain of ADAR1 may prevent ZBP1 activation in an editing-independent manner (Zhang et al., 2022; Fig. 2 B). Alternatively, mutual binding of ZBP1 and ADAR1 to Z-RNA may prevent recruitment of RIPK3 to active ZBP1 (Karki et al., 2021).

Collectively, mouse genetics revealed critical upstream (SETDB1 and ADAR1) or downstream (RIPK1/FADD/caspase-8) brakes on ZBP1 activation that prevent autoinflammation. Whether ZBP1 contributes to human autoinflammatory pathology, however, remains to be determined.

## The role of ZBP1 in cancer

In addition to its well-established roles in antiviral immunity and autoinflammation, ZBP1 has recently been implicated in malignant disease. Necroptosis has been suggested to limit the growth of tumors and to facilitate adaptive immune responses through the release of danger signals and neoantigens (Kroemer et al., 2022; Yan et al., 2022). ZBP1 may therefore contribute to antitumor immunity. Indeed, high expression of ZBP1 in melanoma correlates with tumor infiltration by lymphocytes (Zhang et al., 2022) and better prognosis (Mall et al., 2022), and similar observations were reported for triple-negative breast cancer (Huang et al., 2021). Moreover, in mouse models of colorectal cancer and melanoma, cell death driven by Z nucleic acids and ZBP1 limits tumorigenesis (Karki et al., 2021). However, in low-grade glioma, ZBP1 expression correlates with poor prognosis (Mall et al., 2022). Moreover, although ZBP1 is highly expressed in advanced, necrotic tumors and triggers necroptosis of tumor cells, ZBP1-dependent necroptosis was found to facilitate metastasis in breast cancer models (Baik et al., 2021). It is therefore likely that the role of ZBP1 in cancer varies between tumor types or stages.

Understanding the underlying mechanisms determining beneficial and detrimental effects of ZBP1 in cancer remains an important challenge for future studies, but recent work has begun to provide insight into this question. For example, ZBP1 has been shown to play a role in replicative crisis (Nassour et al., 2023). Replicative crisis is a tumor-suppressive barrier initiated when telomeres become very short and unstable. This then triggers cell death via a process involving autophagy (Nassour et al., 2021). Karlseder and colleagues found that telomeric-repeat-containing RNA (TERRA) transcripts synthesized from dysfunctional telomeres are recognized by a human splice variant of ZBP1 that lacks Zα1 (ZBP1-S, Fig. 1 A; Nassour et al., 2023). This study further suggests that ZBP1-S then engages MAVS to potentiate type I IFN responses ultimately resulting in autophagy-dependent cell death. ZBP1-S thus contributes to a safeguarding mechanism preventing cancer initiation. In contrast, in multiple myeloma, ZBP1 activates IRF3 that, together with IRF4, promotes expression of cell cycle genes and thereby facilitates proliferation of malignant cells (Ponnusamy et al., 2022). It is noteworthy that in multiple myeloma cells, a faster migrating ZBP1 isoform is detectable by Western blot (Ponnusamy et al., 2022), which may correspond to ZBP1-S (Nassour et al., 2023).

The role of ZBP1 in cancer is further determined by interactions with treatments and/or drugs. For example, curaxins exert anticancer effects (Jin et al., 2018) and the second-generation curaxin CBL0137 induces the formation of Z-DNA (Safina et al., 2017; Zhang et al., 2022). CBL0137 thereby triggers ZBP1-dependent necroptosis and, in mouse melanoma models, synergizes with immune checkpoint blockade (Zhang et al., 2022). Other examples of beneficial drug/treatment–ZBP1 interactions in cancer include (i) the flavonoid fisetin that induces ZBP1-dependent necroptosis in ovarian cancer cell lines (Liu et al., 2022); (ii) nuclear export inhibitors that unleash ZBP1 (Jiao et al., 2020; Karki et al., 2021); (iii) vinca alkaloids that together with type I IFN induce cell death in a partially ZBP1-dependent manner (Frank et al., 2019); (iv) CDK1 inhibitors that exert cytotoxic effects by triggering assembly of ZBP1 signaling complexes (Ren et al., 2022); and (v) ionizing radiation that is less effective against ZBP1-deficient tumors (Yang et al., 2021). In the latter case, ZBP1 activation has been proposed to result in a feed-forward loop by inducing the release of mitochondrial DNA (mtDNA) that activates cGAS, which in turn upregulates ZBP1 via type I IFN production (Yang et al., 2021). A recent study suggests another interesting link between ZBP1 and cGAS (Lei et al., 2023a). West and colleagues studied anthracycline chemotherapeutics, particularly doxorubicin, which damage mtDNA, and report that ZBP1—once induced by a first round of cGAS-dependent type I IFN signaling—sequesters cGAS in the cytoplasm, which amplifies cGAS activation. This requires direct protein–protein interaction between cGAS and ZBP1 as well as indirect interaction bridged by nucleic acid. Interestingly, the well-known toxicity of doxorubicin that particularly affects the heart (Vejpongsa and Yeh, 2014) appears to be mediated by this cooperation between cGAS and ZBP1 (Lei et al., 2023a).

## Recent developments and future directions

ZBP1 has been implicated in a wide range of diseases and its beneficial or detrimental roles in most settings require intact Zα domains, with Zα2 being particularly important. This suggests that recognition of Z nucleic acids is central to ZBP1’s biology. However, several fundamental questions remain open in this context.

As discussed earlier, viral RNAs are ZBP1 agonists upon virus infection, although yet-to-be-defined cellular RNAs also contribute to ZBP1 activation in this setting (Maelfait et al., 2017). In autoinflammation, ZBP1 activation by duplex RNA formed by base pairing of endogenous retroviral transcripts (Wang et al., 2020), inverted repetitive elements including IR-*Alu*s (de Reuver et al., 2022; Jiao et al., 2020; Jiao et al., 2022) or inverted short interspersed nuclear elements, and other Z-prone repeats in the 3′ UTRs of IFN-stimulated genes (Zhang et al., 2022) have been suggested; however, direct evidence is lacking at present. Another type of unusual cellular RNAs that activate ZBP1 are telomer-derived TERRA RNAs (Nassour et al., 2023). Moreover, it is tempting to speculate that long dsRNAs arising from overlapping genes transcribed in the opposite direction, which are edited by ADAR1 and are known as cis-NATs (Li et al., 2022), activate ZBP1 in some settings. In addition to Z-RNA, ZBP1 is also activated by Z-DNA. mtDNA was proposed to amplify necroptotic signaling via ZBP1 (Chen et al., 2018) and has since been suggested as a ZBP1 agonist in different cancer settings (Baik et al., 2021; Lei et al., 2023a; Yang et al., 2021) and myocardial infarction (Enzan et al., 2023). mtDNA is also released from mitochondria exposed to oxidative stress and may then trigger inflammatory responses via ZBP1 (Saada et al., 2022; Szczesny et al., 2018). Despite this progress, it is at present unclear which precise sequences, if any, in these nucleic acids are recognized by ZBP1. Defining pathogen-derived and self RNAs and DNAs detected by ZBP1 may be achieved by using techniques such as DNA/RNA immunoprecipitation, crosslink immunoprecipitation (e.g., iCLIP), or nuclease protection. Furthermore, whether (and why) the DNA/RNA regions detected by ZBP1 adopt or are prone to adopt the Z conformation remains open. This may be due to sequence, nucleotide modification, and association (or lack of association) with other proteins or perhaps also metabolites. For example, spermine facilitates DNA recognition by cGAS (Wang et al., 2023) and it would be interesting to test if related mechanisms govern nucleic acid detection by ZBP1. Taken together, defining the identities and properties of the Z-DNAs and Z-RNAs that activate ZBP1 in different cell types and different diseases is an important challenge for future work.

A second area for future investigation is the molecular and structural events following Z-DNA/RNA engagement by ZBP1. Although the structures of both Zα domains have been determined (Ha et al., 2008; Kim et al., 2011b; Schwartz et al., 2001), the structure of full-length ZBP1 is unknown. The structure of ZBP1 predicted by AlphaFold (https://alphafold.ebi.ac.uk; Fig. 1 B) shows Zα1 and Zα2 with high confidence and the RHIMs with intermediate confidence; however, for the majority of the remaining protein, no structure is predicted (Jumper et al., 2021; Varadi et al., 2022). For other sensors such as RIG-I, full-length structures show domain rearrangements upon nucleic acid binding, which revealed important insights into how downstream signaling is initiated (Kowalinski et al., 2011). Such information is lacking for ZBP1. Dimerization of ZBP1 has been suggested to trigger signaling (Wang et al., 2008), and available crystal structures show two Zα domains binding Z-DNA on opposite sides (Ha et al., 2008; Schwartz et al., 2001). Zα1/Zα2 cooperation, ZBP1 dimerization, and/or perhaps the formation of higher-order structures may therefore be important in ZBP1 signaling. Local concentration of ZBP1 either into stress granules (Szczerba et al., 2023) or through 2′-5′ oligoadenylate synthetase-like protein-mediated phase separation (Lee et al., 2023) may promote the generation of multimeric ZBP1 complexes. Future work to understand the structure and the structural and molecular dynamics of ZBP1 is warranted.

A third and related question pertains to how the varied signaling outcomes of ZBP1 activation are coordinated. The association of activated ZBP1 with RIPK1 and RIPK3 can induce proinflammatory signaling and/or promote regulated cell death. Whether these downstream events occur simultaneously, perhaps due to the formation of large signaling complexes encompassing proteins related to different cell death programs (reviewed in Karki and Kanneganti, 2023; Oh and Lee, 2023), or originate from separate signaling complexes is not clear yet. The precise signaling outcome may depend on the relative expression levels of each signaling component, which is likely to vary between cell types. For example, the RHIM of RIPK1 and the proteolytic activity of caspase-8 suppress ZBP1-induced necroptosis, at least in mouse cells (Lin et al., 2016; Newton et al., 2016; Schwarzer et al., 2020; Yang et al., 2020). The possibility that ZBP1 activates IRF3 and thereby induces type I IFNs (Lei et al., 2023b; Nassour et al., 2023; Ponnusamy et al., 2022; Takaoka et al., 2007) should be studied further with a focus on determining whether these are direct consequences of ZBP1 signaling and/or effects of feedback/priming loops. ZBP1 interactions with other factors such as caspase-6 (Zheng et al., 2020), AIM2 and pyrin (Lee et al., 2021), and TRIF (Muendlein et al., 2022; Muendlein et al., 2021) are likely also important in routing ZBP1 signaling. While binding of TRIF to ZBP1 can occur through homotypic interaction between the RHIMs of both proteins, future biochemical and structural work is needed for a more detailed understanding of how ZBP1 associates with caspase-6, AIM2, and pyrin. Ubiquitination of ZBP1 and downstream signaling components may further regulate signaling, although the functional consequences of these events remain unclear and require further characterization (Kesavardhana et al., 2017; Peng et al., 2022). The cell type in which ZBP1 is activated may be an important determinant of signaling. For example, in cortical neurons infected with Zika virus, ZBP1 induces a transcriptional program to change metabolism; this involves the transcription factor IRF1 and *ACOD1* induction (Daniels et al., 2019).

Interestingly, ZBP1 functions that are at least partially independent of nucleic acid binding have recently been discovered. These include restriction of MCMV infection and skin inflammation caused by RIPK1 and ADAR1 deficiency (Jiao et al., 2020; Jiao et al., 2022), cGAS sequestration in the cytoplasm (Lei et al., 2023b), and most notably heatstroke. Lu and colleagues found that heat stress in cells and mice results in ZBP1-dependent cell death, causing some of the heatstroke-related pathology (Yuan et al., 2022). This requires RIPK3 and ZBP1’s RHIMs but not the Zα domains and may be related to ZBP1 aggregation during heat stress. It would be interesting in the future to test if ZBP1 is also activated in other settings of cellular stress.

Loss-of-function of *RIPK3* has recently been identified in a patient suffering from HSV-1 encephalitis (Liu et al., 2023). Induced pluripotent stem cell–derived cortical neurons generated from patient fibroblasts are resistant to HSV-1–induced cell death and sustain increased viral replication. It is possible that a failure to induce ZBP1-RIPK3–mediated cell death underlies the encephalitis phenotype, although RIPK3 may also function downstream of TNF receptor 1 or TLR3 to induce neuronal death and restrict viral replication. In the future, it will be interesting to look for loss-of-function of *ZBP1* in genetically undefined cases of HSV-1 encephalitis or other viral infections. Conversely, gain-of-function mutations in *ZBP1* may underlie human autoinflammatory pathologies.

An important long-term aspiration is the development of therapeutic interventions that activate or inhibit ZBP1. Activators may promote antiviral and antitumor immunity while inhibitors will be beneficial in inflammatory and perhaps also neurodegenerative (Guo et al., 2023) diseases. Indeed, recent work with the curaxin CBL0137 underlines that the former is a promising approach (Safina et al., 2017; Zhang et al., 2022). Conceptually, pharmacological or biological agents could target the Zα domains of ZBP1, the Zα domain-Z-RNA/DNA interaction, conformational changes likely occurring upon activation, and interactions with downstream signaling or scaffolding proteins. Successful screening for and development of ZBP1 inhibitors and activators, as well as identification of disease settings, patient groups that benefit most, and administration routes, will, however, require continued fundamental research efforts. These should focus on the cellular and molecular biology of ZBP1 and on functional differences between cell types.
